# Group I introns are widespread in archaea

**DOI:** 10.1093/nar/gky414

**Published:** 2018-05-18

**Authors:** Eric P Nawrocki, Thomas A Jones, Sean R Eddy

**Affiliations:** 1 National Center for Biotechnology Information, U.S. National Library of Medicine, Bethesda, MD 20894, USA; 2 Howard Hughes Medical Institute, Harvard University, Cambridge, USA; 3 Department of Molecular and Cellular Biology, Harvard University, Cambridge, USA; 4 School of Engineering and Applied Sciences, Harvard University, Cambridge, USA

## Abstract

Group I catalytic introns have been found in bacterial, viral, organellar, and some eukaryotic genomes, but not in archaea. All known archaeal introns are bulge-helix-bulge (BHB) introns, with the exception of a few group II introns. It has been proposed that BHB introns arose from extinct group I intron ancestors, much like eukaryotic spliceosomal introns are thought to have descended from group II introns. However, group I introns have little sequence conservation, making them difficult to detect with standard sequence similarity searches. Taking advantage of recent improvements in a computational homology search method that accounts for both conserved sequence and RNA secondary structure, we have identified 39 group I introns in a wide range of archaeal phyla, including examples of group I introns and BHB introns in the same host gene.

## INTRODUCTION

Group I introns are canonical examples of catalytic RNAs (1,2). They have been found in bacteria, organelles, bacteriophages, and a few eukaryotic nuclear genomes, but none have yet been found in archaea (3–5). Except for a few group II catalytic introns (6), all known archaeal introns are so-called bulge-helix-bulge (BHB) introns (7,8), named for a consensus RNA structure motif processed by the archaeal transfer RNA (tRNA) splicing endoribonuclease (9–11).

Tocchini-Valentini *et al*. (12) suggested that group I introns went extinct in archaea by evolving into simpler BHB introns, by co-opting a tRNA intron endonuclease for splicing and then decaying to lose RNA catalysis. This idea parallels the widely accepted hypothesis that in eukaryotes, spliceosomal introns and the *trans*-acting spliceosomal machinery evolved from catalytic group II introns (13–15). The evolutionary history and phylogenetic distribution of the known types of introns—eukaryotic spliceosomal introns, eukaryotic tRNA introns and archaeal BHB introns, and sporadically distributed group I and group II introns—have long been subjects of intense interest (16–18).

Group I introns might exist in archaea yet have been missed, because they are difficult to identify by sequence analysis (19). They conserve a distinctive catalytic RNA secondary and tertiary structure, but often show little primary sequence conservation, so sequence similarity search methods such as BLAST (20) can fail to detect them. They occur most often in ribosomal RNA (rRNA) genes or tRNA genes, where the presence an intervening sequence is easily recognized in sequence alignments, but distinguishing an unexpected group I from an expected BHB intron in an archaeal host gene requires RNA structure prediction. Many group I introns encode a homing endonuclease gene (HEG) in their RNA sequence, which is responsible for a DNA mobility event that propagates the intron into intronless host genes (5). HEGs can be identified by sequence similarity, but homologous HEGs are also found in many other classes of mobile DNA elements, including archaeal BHB introns, group II introns, inteins, and freestanding mobile HEGs (21,22). Many HEG homologs are known in archaea, and to date have been attributed to one of these other classes of mobile elements.

One way to identify group I introns is to perform a computational search for their conserved RNA structural features. Various computational methods have been developed for searching genome sequences for a combination of RNA structure and sequence similarity (23,24), including some programs specifically designed for group I intron detection (19,25). Perhaps the most sensitive general-purpose method for searching for homologs of a given RNA multiple sequence alignment and consensus RNA secondary structure uses probability models called covariance models (CMs) (26–28). Infernal, a CM software package for RNA homology search and alignment (29), is the basis for the Rfam RNA database, which contains CMs for 2500+ RNA families (30).

CM methods are computationally demanding. Until recently, database-wide Infernal searches for large RNA consensus structures required an infeasible amount of time. Over the past several years, a series of advances have greatly accelerated Infernal. It is now possible to do sensitive large-scale homology searches for consensus RNA structures as large as group I introns (29,31).

Here, we describe using Infernal to identify 39 group I introns in a wide variety of archaeal phyla.

## MATERIALS AND METHODS

Subtype-specific group I intron alignments were downloaded from the GISSD database (46) in October 2014. The IB4 alignment was edited to correct two mistakes in secondary structure annotation, and the IA3 alignment was edited to correct a format issue.

All archaeal sequences in GenBank were retrieved in September 2017 using the Entrez query ‘txid2157[orgn]’ (the NCBI Taxonomy ID number for ‘Archaea’ is 2157) which returned 591 443 sequences comprised of 6 710 959 751 total nucleotides.

Infernal v1.1.2 (29) was used to build and calibrate CMs from group I intron alignments, using the cmbuild and cmcalibrate programs with default parameters. The cmsearch program was used with two different parameter settings to search each of the 15 CMs against the archaeal sequence data, once with default parameters, and once with the command line option --anytrunc which can improve performance on truncated or interrupted sequences, such as group I introns interrupted by embedded HEGs. Hits with an *E*-value ≤0.01 were kept, and overlapping hits were removed, keeping the hit with the lowest *E*-value. Highly similar hits were removed such that no two remaining hits were more than 97% identical given their alignment to the CM.

Homing endonuclease gene (HEG) homologs were detected by translating each of the 39 introns in all six frames and searching all ORFS ≥20aa against Pfam 31.0 (47) using the HMMER 3.1b2 hmmscan program (48), with an *E*-value threshold of ≤10^−3^.

Large subunit (LSU) and small subunit (SSU) rRNA sequences were identified using cmsearch in all sequences with an IB4 or IA3 hit using the Rfam 13.0 (31) archaeal LSU and SSU models (RF01959 and RF02540) with the command line option --rfam, which accelerates searches by enforcing strict sequence-based filters.

To check that host LSU and SSU rRNA sequences were archaeal in origin, we scored each rRNA sequence with the domain-specific Rfam covariance models LSU_rRNA_archaea, LSU_rRNA_bacteria, and LSU_rRNA_eukarya (RF02540, RF02541, RF02543) or the corresponding SSU models (RF01959, RF00177, RF01960) and checked that the highest-scoring alignment was to the archaeal profile. This is only suitable as a coarse-grained check; similarity scores to multiple alignment profiles are a crude substitute for phylogenetic classification on trees.

Taxonomic classification of 25 SSU rRNAs identified on the same contigs as the introns was done on the RDPClassifier web server (49) using training set 16 and default parameters, at a threshold of ≥80% confidence.

## RESULTS AND DISCUSSION

The input to an Infernal search is a multiple RNA sequence alignment with consensus RNA secondary structure annotation. Group I introns have been classified into five types (IA, IB, IC, ID and IE) and further subdivided into fourteen subtypes (IA1, etc.) based on variation in conserved consensus secondary structure (50,51). No single alignment, such as the Rfam database group I intron alignment (Intron_gp1, RF00028), seemed likely to capture this structural diversity well, so we followed the approach of Lang *et al*. (19) and used subtype-specific models. We obtained fourteen subtype-specific alignments of group I introns from the Group I Intron Sequence and Structure Database (GISSD) (46), lightly edited to correct some errors in consensus structure annotation, and built CMs from each of them with Infernal. The IA3 subtype structural alignment, for example, contains 56 sequences, 205–374nt long, with an average pairwise sequence identity of 45%, and an annotated consensus structure of 81 base pairs. The IB4 subtype alignment contains 89 sequences, 203–392nt long, with 44% average pairwise sequence identity, and 71 annotated consensus base pairs.

The quantity and diversity of known archaeal genome sequence has grown rapidly recently, due to metagenomic studies of uncultivated archaea (34,36–44), including representatives of several newly proposed archaeal phyla and superphyla (33,35,52). We obtained a 6.7Gb file of all 591 443 archaeal sequences in GenBank using a taxonomic query of ‘txid2157[orgn]’ at NCBI.

We searched this archaeal sequence data with each of the 14 group I subtype CMs and with the Rfam Intron_gp1 model. Most of the 15 searches take less than 30 minutes on four CPU cores. The IA1 and IC3 subtype alignments include large HEG insertions, and these larger queries take 1–4 h. The Infernal search program, cmsearch, assigns log-odds probability scores to RNA secondary structure alignments and ranks putative hits by a measure of the statistical significance, the *E*-value (expectation value), the estimated number of false positives at that score threshold. After removing overlapping hits and keeping the hit with the lowest *E*-value, there were no significant hits at a threshold of *E* < 0.01 for ten subtypes and the RF00028 model. IA2 and IC3 searches found three and two significant hits, respectively, but upon examination, we were unsure whether these were truly in archaeal sequences, as opposed to misannotated or contaminating bacterial or phage DNA, and we did not consider these further.

The IB4 and IA3 CM searches found a total of 95 significant hits with an *E*-value of 0.01 or less, after we removed overlapping, lower scoring hits. We examined these hits and removed 25 that corresponded to redundant identical sequences in the archaeal database; seven where the sequence record does not contain a complete intron; three where we were not confident that the sequence folded into a complete group I consensus structure; and four where we were not confident that the host gene is an archaeal sequence. Because introns may contain nonconserved insertions including HEGs, a single intron may be identified by Infernal in more than one local alignment piece. After assigning the remaining hits to single introns, we identified a total of 39 nonredundant, full length putative group I introns, 27 IB4s and 12 IA3s. Most were identified by at least one strongly significant hit; 30 of the 39 have at least one hit of *E* < 10^−10^. Table 1 summarizes attributes of these introns, their source sequences, and their locations in their host genes.

**Table 1.** Summary of 39 identified archaeal group I introns showing the name we use to refer to each intron; GenBank accession; phylum assignment (R: inferred by RDPClassifier; G: annotated in GenBank; –: unclassified by either); coordinates of the intron in the source sequence; *E*-value of the most significant Infernal hit to this intron; insertion position in the host ribosomal RNA gene in canonical *E. coli* coordinate numbering (32); and citation for the source sequence. For four phylum assignments, noted by ?, Genbank annotation and RDPClassifier inference were in conflict, and we chose one (see Materials and Methods). For 14 assignments, noted by *, an SSU rRNA was present but RDPClassifier was unable to resolve a phylum-level assignment. IB4.16 is intentionally missing (see text). An expanded table is provided as a parseable .csv file in supplementary data.

Intron	Sequence		Intron	Infernal	Insertion	
Name	Accession	Phylum	Coordinates	*E*-value	Position	Reference
IA3.1	CP010426.1	Woesearchaeota (R)	664815–664111	2.2e–16	LSU/2593	(33)
IA3.2	LQMQ01000054.1	Euryarchaeota* (G)	4017–4815	4.6e–8	LSU/2500	(34)
IA3.3	LQMP01000030.1	Euryarchaeota (G+R)	66091–65273	3.3e–11	LSU/2500	(34)
IA3.4	JWKY01000014.1	–	16576–15805	4.4e–12	LSU/2593	(33)
IA3.5	ASMP01000002.1	Pacearchaeota$^?$ (R)	130592–130914	7.0e–33	LSU/2593	(35)
IA3.6	KP308561.1	Pacearchaeota (R)	16134–15526	2.9e–27	LSU/2593	(33)
IA3.7	KP308720.1	–*	23163–23468	3.5e–42	LSU/2593	(33)
IA3.8	KY476711.1	–*	66905–66301	1.1e–26	LSU/2593	(36)
IA3.9	MNVE01000016.1	Micrarchaeota* (G)	260342–261072	2.2e–15	LSU/2593	(37)
IA3.10	MNVF01000017.1	Micrarchaeota (G)	6638–7348	4.9e–16	LSU/2593	(37)
IA3.11	MNVH01000001.1	Micrarchaeota* (G)	9089–8693	1.6e–36	LSU/2593	(37)
IA3.12	MNVZ01000017.1	Pacearchaeota (G)	14519–14202	2.8e–30	LSU/2593	(37)
IB4.1	CBTY010000008.1	Thaumarchaeota (G+R)	399334–399054	1.0e–34	LSU/1931	(38)
IB4.2	KP308713.1	Woesearchaeota (R)	1026–667	2.3e–32	LSU/1931	(33)
IB4.3	KP308715.1	Woesearchaeota (R)	33361–33696	8.1e–39	LSU/1931	(33)
IB4.4	KP308717.1	Woesearchaeota (R)	8526–8928	3.0e–32	LSU/1931	(33)
IB4.5	KP308748.1	Woesearchaeota (R)	21438–21748	1.4e–25	LSU/1931	(33)
IB4.6	KP308561.1	Pacearchaeota (R)	17157–16694	9.1e–31	LSU/1931	(33)
IB4.7	KP308720.1	–*	21440–21763	1.4e–35	LSU/1923	(33)
IB4.8	AEIX01000219.1	Nanohaloarchaeota (G+R)	2242–1950	7.8e–21	SSU/1498	(39)
IB4.9	BA000048.1	Aigarchaeota$^?$ (G)	1314432–1313300	2.0e–7	LSU/1923	(40)
IB4.10	AEIX01000215.1	Nanohaloarchaeota (G)	10957–11697	4.5e–10	LSU/1931	(39)
IB4.11	AP011903.1	Thaumarchaeota$^?$ (R)	23185–23940	1.0e–6	LSU/1923	(40)
IB4.12	AQRL01000028.1	Woesearchaeota$^?$ (R)	30565–31289	9.2e–17	LSU/1931	(35)
IB4.13	ASPK01000003.1	Thaumarchaeota* (G)	12290–11538	7.5e–5	LSU/1923	(35)
IB4.14	AGCY01000458.1	Euryarchaeota (G+R)	2850–3533	1.1e–11	LSU/1931	(41)
IB4.15	KP308720.1	–*	21772–22514	3.3e–15	LSU/1931	(33)
IB4.17	MNVF01000017.1	Micrarchaeota (G)	5465–5748	2.4e–29	LSU/1923	(37)
IB4.18	MNVH01000001.1	Micrarchaeota* (G)	10442–9750	6.2e–8	LSU/1923	(37)
IB4.19	MNVH01000001.1	Micrarchaeota* (G)	13564–12830	2.7e–16	SSU/1498	(37)
IB4.20	MFRR01000014.1	Micrarchaeota (G)	65096–64410	5.0e–11	LSU/1923	(42)
IB4.21	MNVZ01000017.1	Pacearchaeota (G)	15452–15175	3.0e–27	LSU/1923	(37)
IB4.22	MFWP01000012.1	Pacearchaeota* (G)	2846–3187	5.2e–28	LSU/1931	(42)
IB4.23	MNFA01000040.1	Thaumarchaeota (G+R)	5119–5802	1.9e–10	LSU/1931	(43)
IB4.24	MNVG01000014.1	Micrarchaeota* (G)	9943–10190	1.7e–29	LSU/1931	(37)
IB4.25	MNVJ01000003.1	Micrarchaeota* (G)	25302–24603	6e–14	LSU/1931	(37)
IB4.26	MNVJ01000003.1	Micrarchaeota* (G)	28477–27735	4.3e–17	SSU/1498	(37)
IB4.27	MNWW01000056.1	Woesearchaeota (G+R)	8341–9021	1.3e–10	LSU/1931	(37)
IB4.28	NJDR01000009.1	Aenigmarchaeota (G)	12303–13059	3.5e–10	LSU/1923	(44)

We looked at three other lines of evidence, independent of Infernal similarity scores, that provide additional support for a conclusion that these are group I introns.

**Figure 1.**
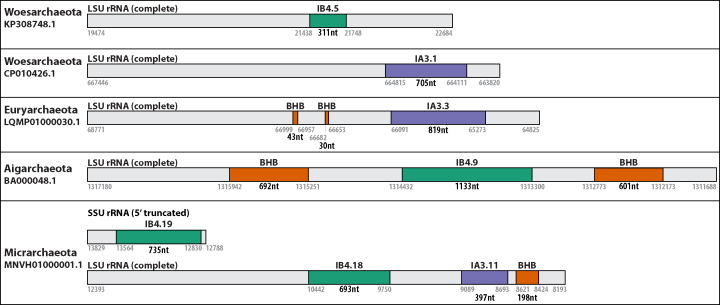
Coordinates of LSU and SSU rRNA host genes, group I introns and BHB introns in five archaeal sequences in GenBank.

First, group I introns are most commonly found in rRNA and tRNA host genes (3), and they tend to be inserted at a small number of conserved homologous positions (32). We identified the host gene for each intron by similarity searches with flanking sequence. All 39 introns are in rRNA genes, 36 in large subunit (LSU) and 3 in small subunit (SSU). Figure 1 shows a schematic of the coordinates of six group I introns in LSU and SSU host genes, sometimes co-occurring with archaeal BHB introns in the same gene. We identified intron insertion positions in canonical *E. coli* coordinate numbering (Table 1). With the exception of the SSU/1498 insertion position, all insertion positions are previously observed insertion sites for group I introns (32).

Second, we looked for evidence of homing endonuclease genes in the putative introns. We translated each intron sequence in six frames and searched all ORFs of length ≥20 against all known Pfam protein domains (47), which include several distinct HEG families (53). Twenty-one of the 39 introns contain ORFs with significant similarity to a Pfam domain, all to LAGLI-DADG homing endonucleases (Pfam LAGLIDADG_1, LAGLIDADG_2, or LAGLIDADG_3; PF00961, PF03161, PF14528).

**Figure 2.**
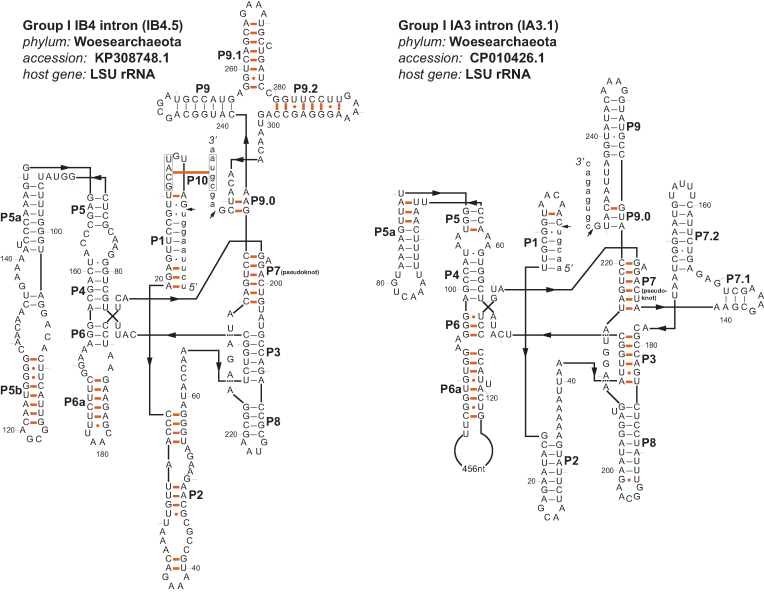
Predicted secondary structures of the IB4.5 and IA3.1 introns. Base pairs shown in thin black lines (or black dots, for GU pairs) are in the Infernal alignments. Those shown by thick red lines (or red dots) were manually predicted. Exonic residues are shown in lower case. Intron positions are numbered starting at 2; by convention, residue 1 is an exogenous added guanosine. Representation follows conventions of Cech *et al*. (45).

Third, the consensus secondary structure identified by an Infernal local alignment is typically just a subset of the base pairs in any individual RNA structure. Infernal is also unable to model pseudoknots, so for the P3/ P7 pseudoknot in the group I intron core, the Infernal models include the P3 helix (because its sequence is less conserved and has more covariation information) and P7 is aligned only as primary sequence. Identifying a P7 helix, other secondary structure elements typical of group I introns, and additional base pairs that extend Infernal-predicted consensus helices provide more confidence in the predicted introns. We manually identified a P7 helix in all 39 predicted introns (Supplementary Table). We manually predicted complete group I intron structures for two introns, IB4.5 and IA3.1. For IB4.5, the cmsearch alignment predicted 58 consensus basepairs; our structure prediction includes 47 of these, and 56 other basepairs. IA3.1 contains a large insertion in P6 with homology to LAGLI-DADG homing endonucleases, so Infernal identified this intron in two local alignment pieces without identifying the P6 and P3 helices that span the insertion. Our structure prediction includes 54 of the 56 base pairs predicted by cmsearch, and 30 additional basepairs including P3 and P6 helices. Figure 2 shows the predicted complete IB4.5 and IA3.1 secondary structures, which are consistent with canonical group I intron structures (2).

The 39 introns are found in 31 different GenBank sequences annotated as archaeal, all of which were obtained by metagenomic environmental sampling. Because GenBank annotation can be unreliable, especially for the phylogenetic source of sequences obtained in metagenomic samples, we also checked that each host rRNA gene sequence scored higher for archaeal SSU and LSU rRNA Rfam consensus models than to bacterial or eukaryotic models. An example of a sequence that failed this check is an LSU rRNA in GenBank MBAA01000200.1, a 4.7 kb contig that was binned into a Lokiarchaeota genome assembly, but appears to be bacterial. We had already named two introns from this contig (IA3.13 and IB4.16) before removing them from our analysis, which is why they are missing in Table 1.

**Figure 3.**
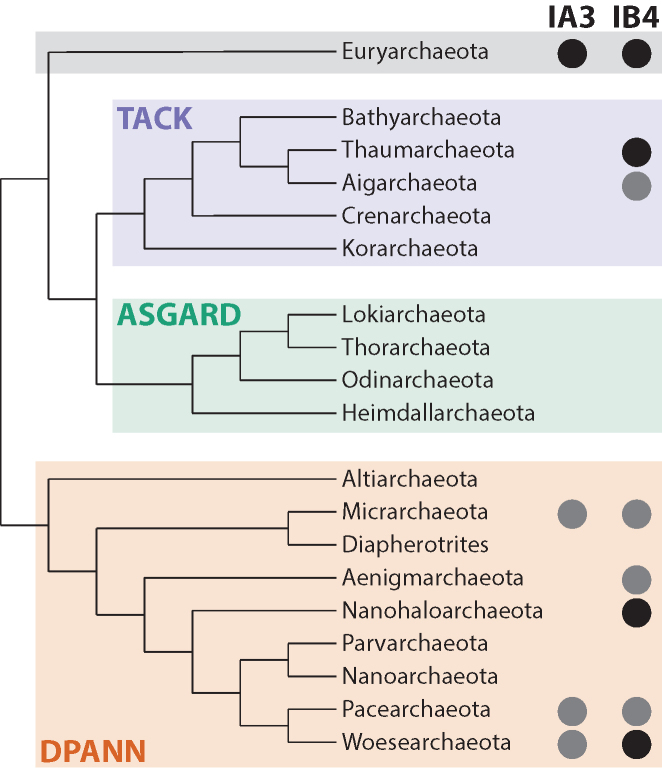
Distribution of identified group I introns in archaeal phyla. Taxonomic assignments of IA3 and IB4 introns from Table 1 are shown on a cladogram of archaeal phyla and superphyla (54). Dark circles indicate occurrence of one or more introns with higher confidence assignments, where GenBank annotation and RDPClassifier agreed.

We asked whether group I introns are broadly distributed across the archaeal phylogeny, or if they only occur in some particular clade. Specific phylum assignments are annotated in 25 of the 31 GenBank records. Both because of the unreliability of these annotations, and also because the taxonomy of archaeal phyla has been expanding (33,35,52), we sought an additional means of assigning sequences to archaeal phyla. SSU rRNA is a convenient and well-established phylogenetic marker for phylum-level classification, much more so than LSU rRNA. Although almost all the introns are in LSU rRNA genes, the genes for SSU and LSU rRNAs are typically adjacent, and we identified an SSU rRNA gene in 25 of the 31 source sequences. The SSU rRNA classification tool RDPClassifier (49) predicts a confident phylum assignment for 16 of these 25 SSU sequences. In four cases, RDP assignments conflicted with GenBank annotation. Three were cases where a new archaeal phylum was proposed after the GenBank sequence was deposited (for which we retained the RDP assignment), and one resulted from a mislabeled training sequence in RDPClassifier (for which we retained the Genbank annotation). Consensus phylum assignments are summarized in Table 1. Figure 3 shows that the introns are distributed widely across archaeal phyla and superphyla, even when counting only cases where GenBank and RDPClassifier phylum assignments strictly agree.

Various sources say that group I introns have not been identified in archaea before (3–5,12,55). However, we do note that in an analysis of an archaeal metagenomic sequencing survey, Nunoura *et al*. (40) referred in passing to an HEG-containing LSU rRNA intron, the same intron that we call IB4.11, as a group I intron. They apparently assumed that any HEG-containing intron is a group I. They did not show evidence to distinguish it from a BHB intron (which often contain HEGs), and they stated that archaeal group I introns were known, citing work on hyperthermophilic bacteria, not archaea (56).

Infernal searches are convenient but not strictly necessary to find these introns. BLAST searches can also identify significant similarities between some of these archaeal intron sequences and some known group I introns in bacteria and eukarya. One reason they have been missed in previous archaeal genome annotations may be that a few informative similarities would be hard to spot amidst a long list of BLAST hits to rRNA and HEG sequences.

The failure to identify group I introns in archaea has previously been attributed to their having evolved into BHB introns and gone extinct (12). It remains plausible that BHB introns derived from group I introns, but our work shows that group I introns are not extinct in archaea, and we identified cases where BHB and group I introns co-exist in the same host gene (Figure 1).

Development of CM-based RNA similarity search methods was originally motivated by a desire to search for group I introns (57), but until recently, these methods were too computationally demanding to systematically search genome sequence data with consensus RNA structures this large (29,31). CM-based homology searches for essentially any structural RNA or RNA element are now feasible.

## Supplementary Material

Supplementary Data
